# In the December 2010 issue

**DOI:** 10.1590/S1980-57642010DN40400001

**Published:** 2010

**Authors:** Ricardo Nitrini

**Affiliations:** Editor-in-Chief

In this issue we have published five reviews and 10 original papers.

**Nitrini** presents a view on the complex issue of the preclinical diagnosis of Alzheimer’s disease, discussing its relevance for the development of preventive treatments. In contrast, for the asymptomatic individual, the knowledge of a high risk of developing Alzheimer’s disease, may be disruptive. A research method to overcome this potential problem is suggested.

**Castro et al. review** the economic impact of Alzheimer’s disease for health systems and society. The total cost of the disease, generated by the sum of direct and indirect cost, is supported by relatives, other informal carers, by social health services and by the public and private health systems. Alzheimer’s disease needs to be considered when designed public health policies for the future, and it is necessary that data on costs are investigated in each South American country, where these data have been scarcely reported, to face the challenges of this disease of increasing prevalent disease.

**Damasceno** reviews the challenges of research on cognitive disorders due to the complexity of functions studied and to the numerous variables implicated. Methodological issues related to confounders such as age, education, size and etiology of lesions, and also to the test and testing conditions are presented and discussed.

**Rodrigues et al.** reviews the evidences on neuroplastic processes resulting from long-term musical training. They review recent findings showing positive effects of musical training on non-musical cognitive abilities, which probably could reflect plastic changes in brains of musicians.

**Spezzano and Radanovic** review the differences between naming of objects or verbs in aphasia and highlight that naming of different semantic and grammatical categories differ in its lexical properties and have distinct neuroanatomical substracts. They conclude that to optimize the rehabilitation efforts and increase its efficacy, more studies with different types of aphasia and the use of naming abilities in different contexts (especially discourse and spontaneous speech), and with different languages, are still necessary.

**Engelhardt et al.** investigated the (pre) fronto-(penduncule)-pontine projection that represents the Arnold’s bundle using diffusion tensor imaging and tractography (DTI-TR). The prefrontopontine segment of the cortico-ponto-cerebellar path, one of the components of the cerebrocerebellar system, was revealed with DTI-TR. This prefrontal segment is anatomically related with the evolutionary newer parts of the cerebellum that participate in the neural circuits underpinning cognitive, emotional, and behavioral control.

**Akanuma et al.** performed a transcultural study comparing two elderly populations of Japanese individuals for the presence of writing errors in Kanji and Kana writing systems. Immigrants from Japan, living in Brazil, and Japanese living in Miyagi, Japan, were compared. Lower levels of education of immigrants and less constant use of the Kanji system may have been responsible for the higher frequency of errors in immigrants.

**Senaha et al.** reported their findings on the rehabilitation for lexical reacquisition in three patients with semantic dementia. Training for lexical reacquisition was based on the principles of errorless learning approach. In spite of the progressive semantic deterioration, reacquisition of lost vocabulary was possible with rehabilitative intervention.

**Costa et al.** report a cross-sectional study of a sample of patients from the Dyslipidemia and High Cardiovascular Clinic, from Southern Brazil, investigating the relationship among obesity, cognitive impairment and depressive symptoms in patients with high cardiovascular risk. Obese patients showed smaller scores in the Mini-Mental State Exam when compared to non-obese patients (p=0.0012). Besides, more severe depressive symptoms were associated with higher likelihood of cognitive impairment.

Wajman and Bertolucci investigated the possible association between education level as well as previous professional occupation and the objective cognitive and functional evaluations in Alzheimer’s disease. In this retrospective study there was indication that not only the total of years of education, but also professional occupation has impact on cognition and functionality in accordance with the hypothesis of cognitive reserve.

**Ribas et al.** evaluated the stress level and performance in attention tests in food handlers, comparing individuals with more than 5 years in this activity with those with less than 5 years. Higher stress level and poorer performance in attention tests were seen in individuals with more than five years of activity.

**Ferretti et al.** investigated the morphometric brain changes during aging in a necropsy series from Brazil. They analyzed data from 414 elderly subjects with mean age of 67.1 years, and found that brain weight and volume (with or without corrections) decrease during aging, in opposition to density, and that these reductions are more pronounced in women.

**Nitrini et al.** present series of cases of neuropsychiatric syndromes caused by neuropsyphilis emphasizing their different forms of presentation and the difficulties they impose to the diagnosis. They emphasize the need of ruling out neurosyphilis in cases with neuropsychiatric symptoms.

**Garavello et al.** compared the evolution of patients with Alzheimer’s disease with and without depressive symptoms. It is conceivable that depressive symptoms lead to a higher functional and cognitive detriment in Alzheimer’s disease, but in this study there were no differences in the evolution of Alzheimer’s disease patients with or without depressive symptoms, although depressive symptoms increased emotional burden of caregivers.

**Castello Branco-Silva and Brucki** reported a case with complex auditory and visual hallucinations probably caused by brainstem lesions and discuss the pathophysiology of these bizarre phenomena.

**Ricardo Nitrini** Editor-in-Chief

